# Villous atrophy in the terminal ileum is a specific endoscopic finding correlated with histological evidence and poor prognosis in acute graft-versus-host disease after allo-hematopoietic stem cell transplantation

**DOI:** 10.1186/s12876-018-0829-4

**Published:** 2018-07-11

**Authors:** Yuusaku Sugihara, Sakiko Hiraoka, Nobuharu Fujii, Shiho Takashima, Yasushi Yamasaki, Toshihiro Inokuchi, Masahiro Takahara, Kenji Kuwaki, Keita Harada, Takehiro Tanaka, Hiroyuki Okada

**Affiliations:** 1 Department of Gastroenterology and Hepatology, Okayama University Graduate School of Medicine, Dentistry and Pharmaceutical Sciences, 2-5-1 Shikata-cho, Kita-ku, Okayama, 700-8558 Japan; 2 Department of Hematology and Oncology, Okayama University Graduate School of Medicine, Dentistry and Pharmaceutical Sciences, 2-5-1 Shikata-cho, Kita-ku, Okayama, 700-8558 Japan; 3 Center for Innovative Clinical Medicine, Okayama University Graduate School of Medicine, Dentistry and Pharmaceutical Sciences, 2-5-1 Shikata-cho, Kita-ku, Okayama, 700-8558 Japan; 4 Department of Diagnostic Pathology, Okayama University Graduate School of Medicine, Dentistry and Pharmaceutical Sciences, 2-5-1 Shikata-cho, Kita-ku, Okayama, 700-8558 Japan

**Keywords:** Acute graft-versus-host disease, Ileocolonoscopy, Terminal ileum

## Abstract

**Background:**

Graft-versus-host disease (GVHD) is a common complication of allo-hematopoietic stem cell transplantation (allo-HSCT). Endoscopic biopsy can provide a definitive diagnosis, but the optimal endoscopic approach for diagnosis remains uncertain. This study evaluated whether ileocolonoscopic imaging can predict acute GVHD severity after allo-HSCT.

**Methods:**

Consecutive patients who underwent allo-HSCT were referred to our institution, and those diagnosed with acute GVHD by pathology were included in this retrospective study.

**Results:**

Fifty-one of 261 patients who underwent ileocolonoscopy were suspected to have acute intestinal GVHD. We performed univariate and multivariate conditional logistic regression with stepwise variable selection; villous atrophy in the terminal ileum remained a statistically significant predictor of GVHD severity (odds ratio, 4.69; 95% confidence interval, 1.07–20.60, *P* = 0.04). Patients were classified into three groups based on ileal endoscopic findings in the terminal ileum: group S, GVHD with severe villous atrophy; group M, mild atrophy; and group N, no atrophy. Compared with patients in groups M and N, those in group S had significant clinical GVHD at diagnosis (*P* = 0.03). In group S, three of four, compared with five of 13 patients in groups M and N, required the addition of second-line agents (*P* = 0.02).

**Conclusions:**

This study showed that severe atrophy of the terminal ileum predicts severe clinical GVHD that is likely to be refractory to steroid treatment. Thus, the severity of terminal ileum atrophy may serve as a tool in predicting clinically severe GVHD.

**Trial registration:**

Trial Registration Number UMIN 000022805, Registration date July 1, 2016.

## Background

Allo-hematopoietic stem cell transplantation (allo-HSCT) has become an essential part of the standard therapeutic repertoire for several haematological diseases, such as acute or chronic leukaemia and other haematological malignancies. Approximately 10−40% of patients who undergo allo-HSCT develop significant clinical acute graft-versus-host disease (GVHD), and the prognosis of steroid-refractory GVHD is poor [1]. GVHD predominantly affects the skin, liver, and gastrointestinal tract after allo-HSCT [2, 3]. For historical reasons, GVHD has been categorized as acute GVHD if it occurs before day 100 after transplantation and as chronic GVHD if it occurs later than day 100 [4]. A condition that arises 100 days after allo-HSCT and shows clinical features of classical acute GVHD is regarded as late-onset acute GVHD [5]. The gold standard for diagnosis is histology (of the skin, liver, gut), and there are well-established criteria for histological diagnosis [6]. However, the optimal endoscopic approach for diagnosis remains uncertain. The treatment of patients diagnosed with acute GVHD cannot be delayed by pathological diagnosis; therefore, acute diagnosis by endoscopic findings has enormous clinical value. Previous studies have reported the macroscopic criteria for acute GVHD or ‘Freiburg Criteria’, including endoscopic features such as spotted erythema or aphthous lesions in the colon and terminal ileum [7] (Table 1). However, the original criteria were created in 1994 and endoscopic technology has advanced since that time; therefore, endoscopic diagnoses should be reconsidered. There have been almost no published reports regarding the relationships between these endoscopic features and the prognosis in acute GVHD or the response to treatment [8].

**Table 1:** The Freiburg Criteria for macroscopic diagnosis of intestinal acute intestinal GVHD

Grade 1	No clear-cut criteria. It suffers to state that there is no GVHD grdade> 2
Grade 2	Spotted erythema, initial aphthous lesions
Grade 3	Aphthous lesions (Crohn-like) or focal erosions
Grade 4	Confluent defects, ulcerations, complete denudation of the mucosa

*GVHD* graft-versus-host disease

With the above in mind, to determine the endoscopic features that aid in the diagnosis of intestinal GVHD, we examined the endoscopic imaging findings of patients with suspected GVHD. In addition, we investigated whether endoscopic features were related to the severity of clinical appearance, responsiveness to treatment, and prognosis.

## Methods

### Patients

This retrospective study included 261 consecutive patients who underwent allo-HSCT and were referred to the Okayama University Graduate School of Medicine between May 2008 and September 2015. We included patients who underwent ileocolonoscopy and had symptoms suggestive of gastrointestinal GVHD, including anorexia, nausea, vomiting, watery diarrhoea, and abdominal pain. Patients were excluded if they had (1) been unable to undergo complete ileocolonoscopy and (2) yielded only unclear images. All patients with diarrhoea underwent a *Clostridium difficile* toxin assay before endoscopy. Demographic information, including the timing of ileocolonoscopy and follow-up data of acute GVHD, disease symptoms, and histological findings were retrospectively obtained from the patients’ medical records. All study participants provided informed consent. The local ethics review committee granted approval for the study (approval 1013), and the study was registered in the University Hospital Medical Network Clinical Trials Registry as UMIN 000022805.

### Endoscopic protocol

**Fig. 1:**
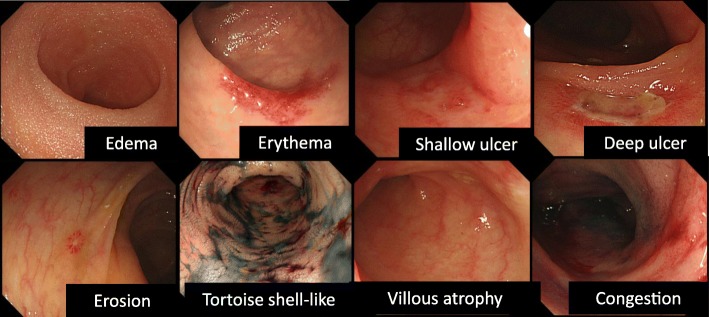
Examples of ileocolonoscopic findings in acute intestinal graft-versus-host disease. Edema: Endoscopic view showed swelling of the entire mucosa. Erythema: Endoscopic view showed spotty redness of the intestinal mucosa. Shallow or deep ulcer: Endoscopic view showed that the tissue of the submucosal layer is exposed and covered with white moss. Erosion: Endoscopic view showed slight scarring of the mucosa. Tortoise shell-like appearance: Endoscopic view with indigo carmine contrast showed sloughing of the mucosa. Villous atrophy: Endoscopic view showed villi of mucosa are low in height. Congestion: Endoscopic view shows conspicuous redness due to blood stasis

Experienced endoscopists, who had performed more than 1000 colonoscopies, performed colonoscopies (Olympus Tokyo) in all patients. Endoscopic images were evaluated based on findings of edema, erosion, erythema, tortoise shell-like appearance, shallow or deep ulcers, congestion, and villous atrophy (Fig. 1) in the terminal ileum, right hemi-colon (cecum, ascending colon, transverse colon), left hemi-colon (the descending colon, sigmoid colon), and rectum. The endoscopists recorded their interpretations immediately after the ileocolonoscopies. Regarding the preparation for ileocolonoscopy, we determined that all patients should receive polyethylene glycol (PEG) if oral ingestion was possible. During ileocolonoscopy, the biopsy protocol was performed in each of the following segments, regardless of the presence or absence of abnormalities that might be indicative of intestinal GVHD: the terminal ileum, right hemi-colon, left hemi-colon, and rectum.

**Fig. 2:**
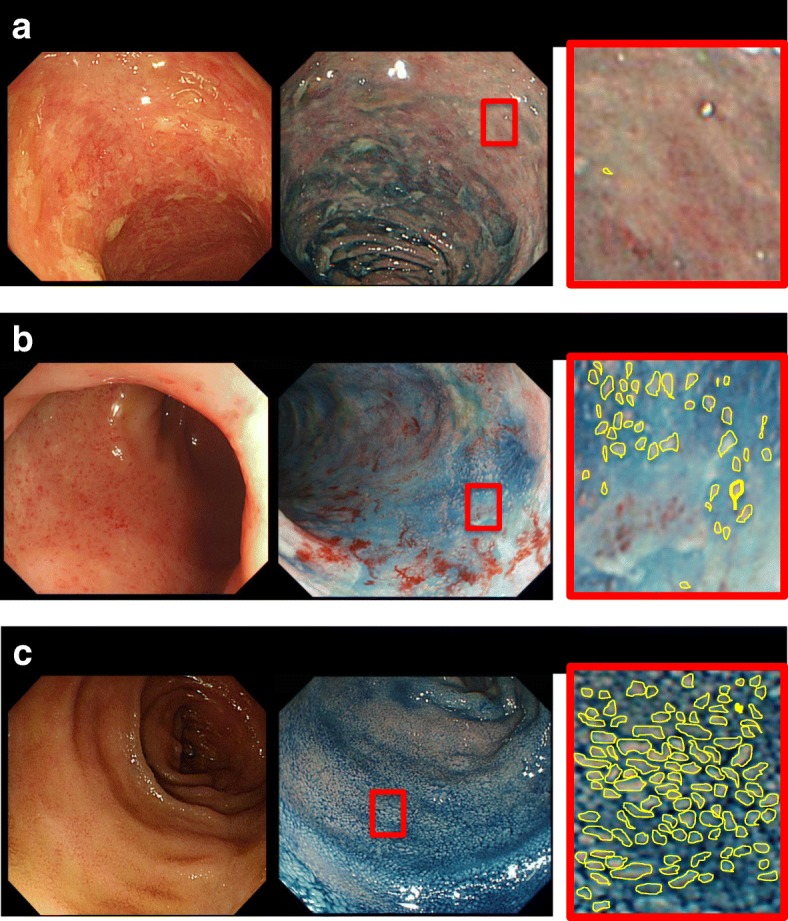
Examples of grading atrophy in the terminal ileum using ileocolonoscopy. Two examples are presented for each grade. Enlarged images of the area denoted with red squares are provided for each group. **a** group S: the endoscopist could not follow the outline (yellow line) of the villi depicted in the white or indigo carmine scattering image at all. Group M: the same as group S. **b** group M: the number of evaluators following the line was more than group S and less than group N. **c** group N: the endoscopist could clearly follow the outline (yellow line) of the villi depicted in the white or indigo carmine scattering image

Characteristic images of graded atrophy severity in the terminal ileum are depicted in Fig. 2. Endoscopic images in the terminal ileum were evaluated by an endoscopist (Y.S. or K.H.), who was not present in the endoscopy room during the examination and was blinded to the endoscopic images. The determination of the grade of atrophy severity in the terminal ileum was evaluated by two endoscopists (Y.S., K.H.). The endoscopic images were screened for signs of the presence of villous atrophy in the terminal ileum, with access to the endoscopic images, as follows: group S = the endoscopist could not follow the outline of the villi depicted in the white or indigo carmine scattering image at all; group *N* = the endoscopist could clearly follow the outline of the villi depicted in the white or indigo carmine scattering image; group M = the number of evaluators following the line was more than that in group S and less than that in group N. Image evaluation was made directly after the screening by an endoscopist who did not perform the examination. All images were evaluated by two endoscopists to check for interobserver agreement. The objective of the present study was evaluation of images from first-time ileocolonoscopy only.

### Pathological and clinical examination

In the present study, the pathological diagnosis of GVHD was based on the following criteria: first, the presence of apoptotic bodies in the epithelium of the ileum, colon or rectum; second, crypt abnormalities of degeneration, dilatation, abscesses or loss; and third, all histological findings determined by haematoxylin and eosin staining and CD8 immunostaining (Table 2). In addition, biopsy samples were immunohistochemically stained for cytomegalovirus. If pathological findings met these criteria in any one of the biopsy samples collected during the ileal endoscopy, the patient was diagnosed with pathological GVHD.

**Table 2:** Criteria of pathological GVHD

1) Histological findings of apoptotic bodies in the epithelium of ileum, colon or rectum.	
2) Crypt abnormalities of degeneration, dilatation, abscesses or loss.	
3) All histological findings determined by HE staining and CD8 immunostaining.	

*GVHD* graft-versus-host disease, *HE* hematoxylin and eosin

Daily stool volume and frequency were measured as the average of 3 d before preparation for endoscopy. The grade of GVHD was determined according to the guidelines of the British Society for Bone Marrow Transplantation [9]. An International Bone Marrow Transplant Registry survey confirmed that most centres considered patients to have steroid-refractory GVHD after 5 d of treatment [10]. Therefore, we defined steroid-refractory GVHD as requiring continuous administration of steroids for 5 d or exhibiting worsening of symptoms within 3 d, or by a haematologist’s determination of whether steroid-refractory GVHD was present. All patients were followed-up for 30 months from the time of endoscopy, and in the case of death, a haematologist made an assessment as to whether the death was attributable to GVHD or another cause.

### Statistical analysis

All analyses were performed using JMP version 11.2 (Stata, College Station, TX, USA). The data were analysed using Student’s *t*-test, Fisher’s exact test, the Jonckheere-Terpstra test, the Cochran-Armitage test, and the Mann-Whitney *U*-test. *P*-values < 0.05 were considered statistically significant. Survival analysis was performed using the Cox proportional hazards model [11] using known or suspected covariates in stepwise multivariate analysis. The reproducibility coefficients were analysed using the kappa agreement coefficient. Interobserver agreement values were classified as follows: poor (≤ 0.20), slight (0.21–0.40), moderate (0.41–0.60), substantial (0.61–0.80), and excellent (0.81–1.00).

## Results

### Patient demographics

**Fig. 3:**
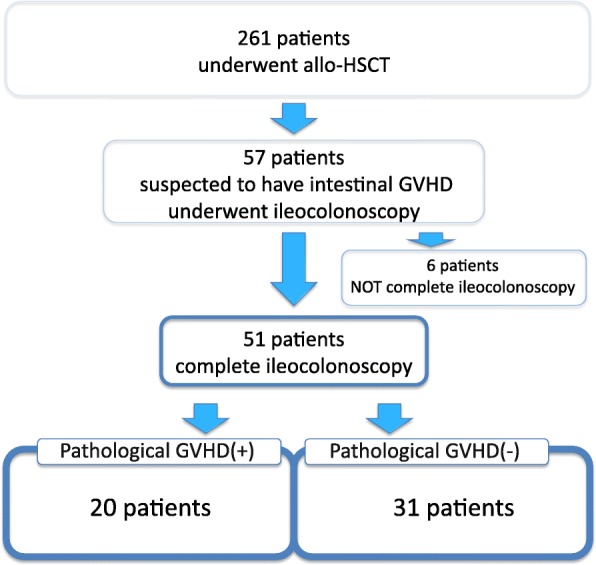
Study flow diagram allo-HSCT, allo-hematopoietic stem cell transplantation; GVHD, graft-versus-host disease

Fifty-seven of 261 patients underwent ileocolonoscopy under the suspicion of acute intestinal GVHD, with symptoms suggestive of gastrointestinal GVHD, including anorexia, nausea, vomiting, watery diarrhoea, and abdominal pain. Six patients were unable to complete the ileocolonodoscopy because of incomplete preparation or unbearable pain and were not included in the study (Fig. 3). The characteristics of these 51 patients are presented in Table 3. Most of the patients were adults, and the youngest was 10 years old. The most common diagnosis was acute myeloid leukaemia. And most frequent stem cell source was bone marrow transplantation. For grade 1 to 4 in Freiburg Criteria, cases were 7, 6, 15, 23 respectively. Almost all recipients of an allogeneic transplant received standard short-term therapy with methotrexate and calcineurin inhibitors for prophylaxis against GVHD. The total number of biopsies performed in 51 patients was 100, including 27 in the terminal ileum, six in the cecum, 13 in the ascending colon, 13 in the transverse colon, six in the descending colon, 19 in the sigmoid colon, and 16 in the rectum. A definitive pathological diagnosis of acute GVHD was confirmed in 20 patients, and 31 patients were not diagnosed with acute GVHD pathologically. Seven of 20 patients were diagnosed as having GVHD concomitant with cytomegalovirus (CMV) infection and two were diagnosed as having GVHD concomitant with thrombotic microangiopathy. *C. difficile* toxin assay results were negative in all patients.

**Table 3:** Characteristics of patients who underwent colonoscopy on suspicion of gastrointestinal GVHD

Total	51
Mean age (range), years	46 (10–67)
Male/female	31/20
Disease requiring transplantation	
Acute myeloid leukaemia	15
Myelodysplastic syndromes	10
Acute lymphoblastic leukaemia	7
Adult T-cell leukemia/lymphoma	4
T lymphoblastic leukemia/lymphoma	3
B lymphoblastic leukemia/lymphoma	3
Chronic myelogenous leukaemia	3
Diffuse large B-cell lymphoma	1
Myelofibrosis	1
Non-Hodgkin lymphoma	1
Extra nodal NK/T-cell lymphoma	1
Polycythaemia	1
Severe combined immunodeficiency	1
Stem cell sources	
Bone marrow	37
Cord blood	8
Peripheral blood stem cell	6
Freiburg Criteria	
Grade 1	7
Grade 2	6
Grade 3	15
Grade 4	23
Pathological diagnosis	
Non-GVHD	31
GVHD	11
GVHD concomitant with CMV	7
GVHD concomitant with TMA	2

*GVHD* graft-versus-host disease, *CMV* cytomegalovirus, *TMA* thrombotic microangiopathy

### Ileocolonoscopic evaluation

The 51 patients who underwent complete ileocolonoscopy were evaluated for a median of 65 d (range, 27−1263 d) after transplantation. Endoscopies were performed in 40 (78%) patients within 100 days after allo-HSCT. Thirty-eight patients (75%) received PEG, eight patients (15%) received glycerine enema, and five patients (9.8%) received no preparation. A definitive pathological diagnosis of acute GVHD was confirmed in 20 patients. Thirty-one patients were not diagnosed with acute GVHD pathologically. For the group diagnosed with GVHD and the group not diagnosed with GVHD, villous atrophy (*P* = 0.002), erythema (*P* = 0.02) in the terminal ileum, and erosion (*P* = 0.02) in the right hemi-colon were significantly related to acute GVHD in univariate analysis. In multivariate analysis using these factors, villous atrophy in the terminal ileum remained a statistically significant factor (odds ratio, 4.69; 95% confidence interval, 1.069−20.597, *P* = 0.04) (Table 4). Most patients with GVHD (14/20, 70.0%) had villous atrophy in the terminal ilium, reflecting a significant association. The overall accuracy, sensitivity, specificity, positive predictive value, and negative predictive value for the utility of villous atrophy on ileocolonoscopic imaging for the diagnosis of GVHD were 70.0, 71.8, 60.9, and 78.6%, respectively.

**Table 4:** Endoscopic findings for diagnosis as GVHD: univariate and multivariate analysis in patients with pathological GVHD positive and negative

Univariate analysis						Multivariate analysis			
		Pathological GVHD(+)		Pathological GVHD(−)		*P*-value	OR	95% CI	*P*-value
		*n* = 20		*n* = 31					
Part	Endoscopic findings	n	(%)	n	(%)				
Terminal	Villous atrophy	14	75	9	29	0.002	4.69	1.07–20.60	0.04
ileum	Edema	4	20	6	19.4	> 0.999			
	Erythema	7	35	2	6.5	0.02	4.67	0.74–29.47	0.10
	Shallow ulcer	1	5	0	0	0.39			
	Deep ulcer	1	5	0	0	0.39			
	Erosion	6	30	4	12.9	0.16			
	Tortoise shell-like	0	0	1	3.2	> 0.999			
	Congestion	0	0	0	0	–			
Right	Edema	13	65	18	58.1	0.77			
hemi-colon	Erythema	9	45	13	41.9	> 0.999			
	Shallow ulcer	3	15	1	3.2	0.29			
	Deep ulcer	1	5	0	0	0.39			
	Erosion	9	45	4	12.9	0.02	1.78	0.35–9.16	0.49
	Tortoise shell-like	0	0	1	3.2	> 0.999			
	Congestion	0	0	0	0	–			
Left	Edema	11	55	15	48.4	0.78			
hemi-colon	Erythema	6	30	8	25.8	0.76			
	Shallow ulcer	2	10	2	6.5	0.64			
	Deep ulcer	2	10	0	0	0.15			
	Erosion	5	25	5	16.1	0.49			
	Tortoise shell-like	3	15	1	3.2	0.29			
	Congestion	0	0	0	0	–			
Rectum	Edema	6	30	11	35.5	0.77			
	Erythema	5	25	5	16.1	0.49			
	Shallow ulcer	2	10	0	0	0.14			
	Deep ulcer	0	0	0	0	–			
	Erosion	1	5	1	3.2	> 0.999			
	Tortoise shell-like	1	5	0	0	0.39			
	Congestion	0	0	0	0	–			

*CI* confidence interval, *OR* odds ratio

### Villous atrophy in the terminal ileum and clinical presentation

Patients were separated into three groups according to villous findings in the terminal ileum: group S, patients with severe villous atrophy or loss (*n* = 6); group M, patients with mild villous atrophy (*n* = 8); and group N, patients with no villous atrophy (*n* = 6). In the interobserver agreement analysis of severity of villous atrophy in all patients, the interobserver kappa coefficient was 0.80 (95% CI 0.75–0.85).

Table 5 shows the characteristics of GVHD patients with villous atrophy. No statistically significant difference was noted between GVHD patients with severe villous atrophy in the terminal ileum and those with mild or no villous atrophy at diagnosis based on age, sex, and days from transplantation (Student’s *t*-test). In group S, three of six patients had stool volumes > 1.5 L/day, with a mean frequency of diarrhoea of 14 events per day. In group M, four of eight patients had stool volumes > 1.5 L/day, with a mean frequency of diarrhoea of 7.5 events /day. In group N, one of six patients had a stool volume > 1.5 L/day, with a mean frequency of diarrhoea of 5.0 events /day. Compared with patients in groups M and N, patients in group S had significantly higher frequency of diarrhoea (*P* = 0.03) (Student’s *t*-test).

**Table 5:** GVHD with villous atrophy in the terminal ileum and treatment response

	Group S	Group M	Group N	*P*-value	*P*-value	
	(*n* = 6)	(*n* = 8)	(*n* = 6)	(for trend)	Group S versus M + N	Group S + M versus N
Mean age (range), years	30 (10–61)	52 (38–58)	57 (33–66)	0.07^a^	0.08^a^	0.15^a^
Sex male/female	3/3	5/3	3/3	> 0.99^d^	1.54^e^	1.54^e^
Days from transplantation, median (range), days	49 (27–231)	69.5 (25–1263)	156.5 (49–1162)	0.47^a^	0.45^a^	0.91^a^
Frequency of diarrhea (times per day), median (range)	14.5 (6–30)	7.5 (0–12)	5.0 (0–8)	0.03^a^	0.02^a^	0.23^a^
GVHD clinical grade Stages 1/ 2/ 3/ 4	0/ 1/ 2/ 3	2/ 0/ 2/ 4	2/ 2/ 1/ 1	0.09^b^	0.75^c^	0.09^c^
Refractory GVHD	5	3	1	0.02^d^	0.10^e^	0.10^e^
Mortality (per person-year)	0.44	0.53	1.05	0.69^f^	0.76^f^	0.80^f^

Group S, GVHD with severe atrophy of villi in the terminal ileum; group M, GVHD with mild atrophy; group N, absence of atrophy of villi; NS, not significant. ^a^, Student’s t test; ^b^, Jonckheere-Terpstra test; ^c^, Mann-Whitney U test; ^d^, Cochran-Armitage test; ^e^, χ2 test; ^f^, log-rank test

### Villous atrophy in the terminal ileum and treatment response

Patients with severe villous atrophy in the terminal ileum (group S) were more likely to present with a severe clinical grade of GVHD than those in groups M and N, although there was no significant difference (*P =* 0.09) (Jonckheere-Terpstra test). Patients in group S were also more likely to require treatment for steroid-refractory disease. In group S, five of six patients (compared with four of 14 patients in groups M and N), required treatment for steroid-refractory disease (*P* = 0.02) (Cochran-Armitage test).

### Villous atrophy in the terminal ileum and mortality

Four of six patients (66%) in group S died within 20 months of allo-HSCT. The mortality rates after allo-HSCT were 0.44 per person-year in group S, 0.53 per person-year in group M, and 1.05 per person-year in group N. There was no difference in all-cause mortality when comparing group S with the other groups (log-rank test). All deaths were attributable to the progression of GVHD itself in the presence of persistent GVHD.

## Discussion

In the present study, we evaluated a total of 51 patients who underwent ileocolonoscopy after allo-HSCT. Among them, 20 patients were pathologically diagnosed with acute GVHD, and we attempted to determine whether atrophy in the terminal ileum was an effective ileocolonoscopic indicator of patient outcomes. Previous reports have shown that ileocolonoscopic evaluation with histological biopsy examination is required to diagnose GVHD. A definitive diagnosis of GVHD is based on the presence of apoptosis, a histological finding indicative of GVHD [12]. However, early and accurate diagnosis and severity assessment of GVHD are critical for successful treatment [13]. Villous atrophy in the terminal ileum has been reported to occur in acute intestinal GVHD and to have high specificity for the disease [7]. However, previous studies have been descriptive and lacking the design or power to analyse the role of ileocolonoscopic grading in GVHD diagnosis. Additionally, while villous atrophy in the terminal ileum is a commonly noted finding, its clinical significance and prognostic value have remained uncertain. To our knowledge, this study is the first to investigate whether ileocolonoscopic findings can predict the clinical severity of GVHD.

Prior studies reported acute GVHD rates of 10–40% in patients with intestinal complaints after allo-HSCT [1, 12]. In the present study, the incidence of acute GVHD was relatively low (7%, 20/261). We assume that the reason for the low incidence of acute GVHD is that only those cases that required ileocolonoscopy were identified. If these patients had undergone upper gastrointestinal endoscopy, there might have been a slight increase in this incidence. Furthermore, using histological criteria for diagnosing GVHD, we documented definitive GVHD by the presence of apoptosis on biopsy examination in all cases.

In a previous study, endoscopic and histological findings on distal colonoscopy were clinically significant for the diagnosis of patients with intestinal GVHD, who were already in very poor general condition and at risk of endoscopy-related complications [14]. However, in this study, we assessed the clinical value of endoscopic findings in the proximal colon and terminal ileum for the diagnosis of intestinal GVHD. The reason for this difference is that in the present study, many patients underwent preparation with PEG, allowing us to more clearly evaluate the proximal colon, and even the terminal ileum. In the present study, 75% (15/20) of patients in the pathological GVHD-positive group and 54% (17/31) of patients in the negative group had various colonoscopic findings, and there was no statistically significant difference between the two groups. Therefore, if colonoscopic examination is limited to the rectum, it is impossible to diagnose GVHD, the severity of GVHD, and steroid-refractory GVHD. In the past, it was thought that inserting the colonoscopy into the terminal ileum often made the procedure uncomfortable for the patient. However, recent developments have made it possible to insert the colonoscopy to the distal end of the ileum in almost all patients following preparation with PEG.

In the present study, we evaluated the extent and severity of villous atrophy in the terminal ileum using ileocolonoscopy, and assigned GVHD cases a clinical grade. First, we found that villous atrophy was evident in acute GVHD using ileocolonoscopy, which confirms previous reports that cases of acute GVHD frequently present with this finding. Second, we observed villous atrophy in 14 (70%) of 20 patients pathologically diagnosed with acute GVHD. Thus, we found that villous atrophy in the terminal ileum is a relatively common finding in these patients and may be effective in diagnosing patients with acute GVHD.

In this study, the severity of villous atrophy in the terminal ileum appeared to be associated with more severe clinical stages of GVHD and correlated with steroid-refractory GVHD, with a statistically significant trend toward a higher frequency in patients with severe villous atrophy. GVHD patients who are steroid-refractory are expected to have a poor prognosis [15]. This study is the first to report that villous atrophy in the terminal ileum specifically predicts steroid-refractory GVHD. Five patients with these findings showed a significant trend compared with those from the other groups. The use of second-line agents was predicted in these patients.

Survival after allo-HSCT depends on many factors, including infection, GVHD, and recurrence of malignancy [15]. Three of the six patients with severe villous atrophy in the terminal ileum at the time of diagnosis died from GVHD or infection in the presence of persistent GVHD within 13 months. In contrast, all patients with no villous atrophy in the terminal ileum at the time of diagnosis survived during the 15-month follow-up. The mean interval to mortality was 198 d in patients with severe atrophy, compared with 247 d in patients with no atrophy, which was indicative of an initially aggressive form of GVHD.

To our knowledge, this study is the first to attempt to correlate the endoscopic finding of villous atrophy in the terminal ileum with the prognosis of GVHD after allo-HSCT. A previous study showed that transplant-related mortality is correlated with colonic crypt loss [16]. However, we believe that endoscopic diagnosis may be more effective than histological diagnosis because endoscopic diagnosis is made earlier.

The present study has several limitations. First, because this was a retrospective study, grading of the terminal ileum was limited to the endoscopic images available. Second, because the data were collected retrospectively, the biopsy location and the number of samples were different depending on the operator performing ileocolonoscopy. Even if endoscopic findings were present at the terminal ileum, whether to perform a biopsy was at the discretion of the operator. A future prospective study with a protocol for the location and number of biopsies is required. Third, in the present study, we tested for *C. difficile* infection and stained for CMV in all patients. However, not all other infections, including adenovirus, enterovirus, rotavirus, and norovirus infections, were examined in all patients. Therefore, we cannot rule out the possibility that these infections influenced the results.

## Conclusions

The results of this study demonstrated that the severity of villous atrophy in the terminal ileum could serve as a marker for severe clinical complications following allo-HSCT. This finding predicted severe diarrhoea and high-clinical-grade GVHD at the time of diagnosis. Furthermore, it predicted GVHD that is more likely to be refractory to conventional steroid treatment and lead to mortality. Future prospective controlled studies are required to confirm our findings and to explore whether a more aggressive treatment approach is needed in patients with severe villous atrophy in the terminal ileum.

## Abbreviations

allo-HSCT: Allo-hematopoietic stem cell transplantation
CMV: Cytomegalovirus
GVHD: Graft-versus-host disease
PEG: Polyethylene glycol
